# Axillary nodal metastasis of operated gallbladder carcinoma: remote site of aggression—a case report

**DOI:** 10.1186/s12893-022-01477-3

**Published:** 2022-01-15

**Authors:** Manu Vats, Lovenish Bains, Pawan Lal, Shramana Mandal

**Affiliations:** 1 Department of Surgery, Maulana Azad Medical College, New Delhi, India; 2 Department of Pathology, Maulana Azad Medical College, New Delhi, India

**Keywords:** Gallbladder, Carcinoma, Oncology, Metastasis, Axilla, Surgery

## Abstract

**Background:**

Gallbladder cancer is a very aggressive type of biliary tract cancer. The only curative treatment is complete surgical excision of the tumour. However, even after surgery, there is still a risk of recurrence of the cancer.

**Case presentation:**

A 63-year-old gentleman presented with the complaint of a non-healing ulcer at upper abdomen for the last 1 month. He had undergone a laparoscopic cholecystectomy at a private centre 4 months ago. Investigations confirmed the diagnosis of epigastric port site metastasis from a primary from gall bladder adenocarcinoma. After undergoing completion radical cholecystectomy with wide local excision of the epigastric ulcer, he received 6 cycles of concurrent chemoradiotherapy. Eighteen months later, he presented to us with bilateral axillary swellings. Investigations confirmed isolated bilateral axillary metastasis and the patient underwent a bilateral axillary lymphadenectomy (Level 3). However, PET scan after 6 months showed widespread metastasis and the patient succumbed to the illness 1 month later.

**Conclusion:**

Axillary metastasis probably occurs due to the presence of microscopic systemic metastasis at the time of development of port site metastasis. An R0 resection of the malignancy is the only viable option for effective therapy. The present case highlights the rare involvement of isolated bilateral axillary lymph nodes as a distant metastatic site with no evidence of disease in the locoregional site. However, the prognosis after metastasis remains dismal despite multiple treatment modalities.

## Background

India is a high incidence area for gallbladder carcinoma (GBC). It is one of the three leading cancers among women of North and North-east India [1]. The age standardized rate (ASR) for GBC in women of North India is 11.8/100,000 population and for north-east India is 17.1/100,000 population [2]. These rates are similar to those seen in Bolivia (14/100,000) and Chile (9.3/100,000) and higher than that found in other parts of Asia: Thailand (7.4), South Korea, Nepal (6.7) and Bangladesh (5.1) per 100,000 population [3]. The average age-adjusted rate among women has risen from 6.2/100,000 in 2001–2004 to 10.4 / 100,000 in 2012–2014 [4]. This data is from 30 population-based cancer registries from all over India, which were set up by the Indian Council of Medical Research (ICMR) [2].

Gallbladder cancer is the most common and most aggressive type of biliary tract cancer with overall 5-year survival rate of only 19% [5]. Owing to their aggressive nature; the only hope of cure remains when the patient presents in an early stage of the disease and complete resection of the cancer is possible. Unfortunately, only 10% of the patients of gall bladder cancer benefit from surgical resection with a curative intent. The rest 90% of the patients present when the disease is advanced, where other modalities of treatment may only palliate the disease [6]. The usual sites of recurrence are the liver and peritoneum [7]. Several cases of port site metastasis from an incidentally discovered gallbladder malignancy after laparoscopic cholecystectomy have been described [8, 9]. However, axillary lymph node metastasis as a presentation of gallbladder cancer metastasis is a very rare occurrence. We present a rare case of bilateral axillary metastasis of gallbladder port site adenocarcinoma with no other initial systemic metastasis.

## Case presentation

A 63-year-old gentleman presented to the surgery department with complaint of a non-healing 3 × 3 cm ulcer at the epigastrium for last 6 weeks. The patient had undergone laparoscopic cholecystectomy four months back at a private hospital for symptomatic cholelithiasis. Cholelithiasis manifested as right upper abdominal pain for 6 months and was confirmed using ultrasonography. The patient was a farmer by occupation and did not consume alcohol and did not smoke. He consumed a mixed diet. There was no history of any malignancy or malignancy related deaths in the family. The histopathology report of the laparoscopic cholecystectomy specimen available with the patient was suggestive of gallbladder adenocarcinoma. The operating surgeon had referred the patient to a tertiary care centre for appropriate management. However, the discharge summary from the private centre did not mention which port site was used for removal of the gallbladder or whether any type of retrieval bag was utilized during the surgery. Contrast Enhanced Computed Tomography (CECT) of the abdomen showed an irregular heterogeneous soft tissue lesion in the anterior abdominal wall of size 3.5 × 3 cm in the epigastric region. There was no lesion in the gall bladder fossa. A positron emission tomography (PET) highlighted a fluorodeoxyglucose (FDG) avid lesion in the epigastrium only, with no uptake in the gall bladder fossa. Incisional biopsy from the epigastric mass revealed moderately differentiated adenocarcinoma with lymphatic invasion. A working diagnosis of epigastric port site metastasis from a primary from gall bladder adenocarcinoma was kept (TxNxM1). He underwent a completion radical cholecystectomy, which included hepatoduodenal ligament clearance and wedge resection of the liver. Wide local excision of the epigastric port site ulcer and mass was also done. The closure of the defect was carried out with sub-lay placement of mesh and abdominal wall rotation flap. The umbilical port site was also excised. Histopathologic examination of the excised epigastric port site specimen reported moderately differentiated adenocarcinoma reaching up to the skin. Two of the four margins (right and inferior) were microscopically involved, and the deep resected margin was free of tumour (R1 resection). The resected hepatic segment showed only focal areas of macro-vesicular steatosis with periportal mild chronic inflammation and none of the lymph nodes were positive for malignancy. Patient made an uneventful recovery. After retrieval of the histopathology report, we had discussed the need for a revision surgery for excision of the margins, however did not consented for a re-excision. An oncology consultation was sought, and after a thorough discussion with the medical oncologist, the patient was planned for concurrent chemoradiotherapy. He received 6 cycles of concurrent chemoradiotherapy (Gemcitabine and Cisplatin; Radiotherapy total of 50.4 Gy in 1.8 Gy per fraction) at our centre. The patient remained asymptomatic on follow up visits during the next 12 months with no evidence of locoregional recurrence.

**Fig. 1:**
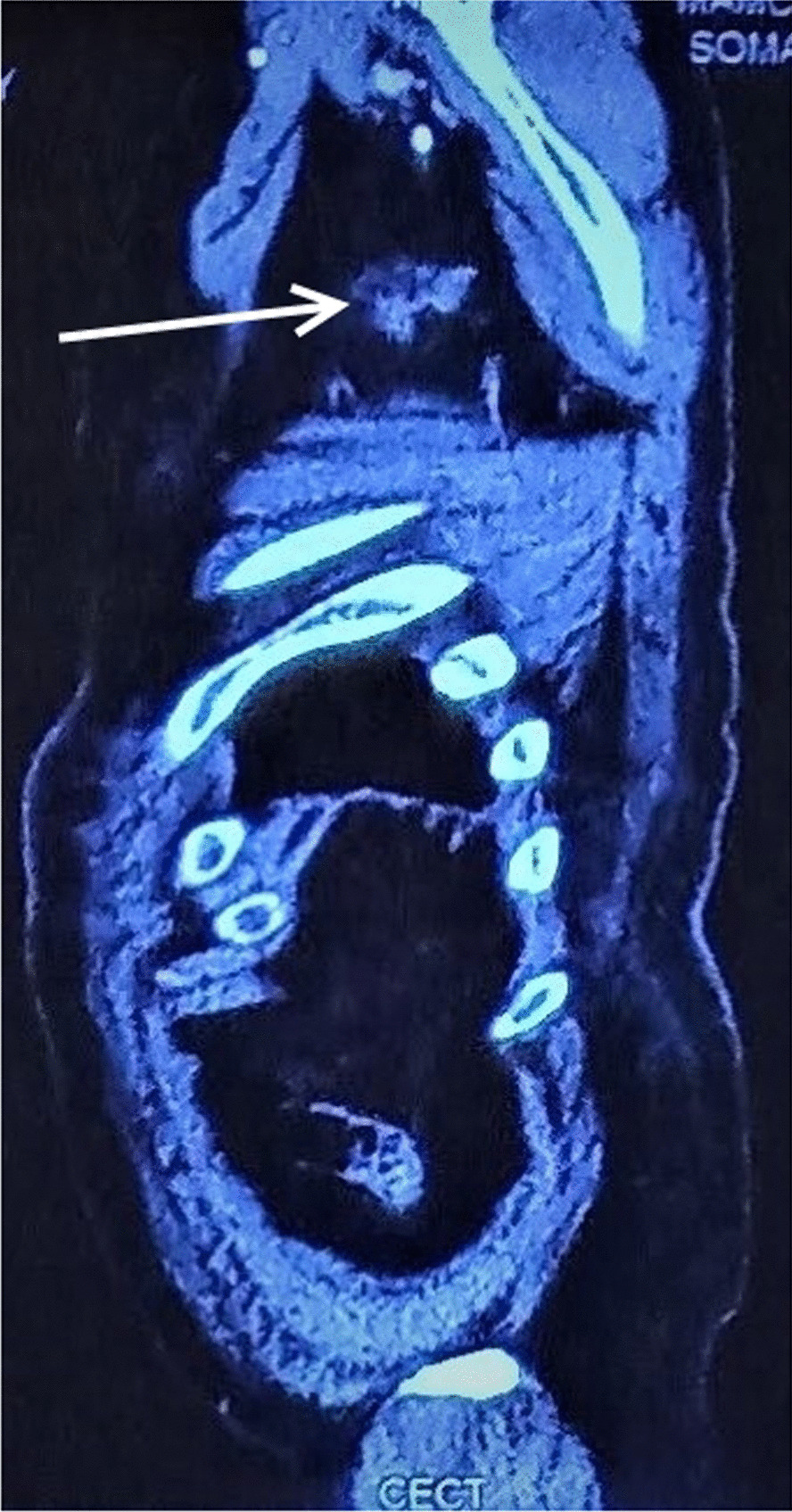
CECT Sagittal section showing left axillary lymph node metastasis (white solid arrow)

**Fig. 2:**
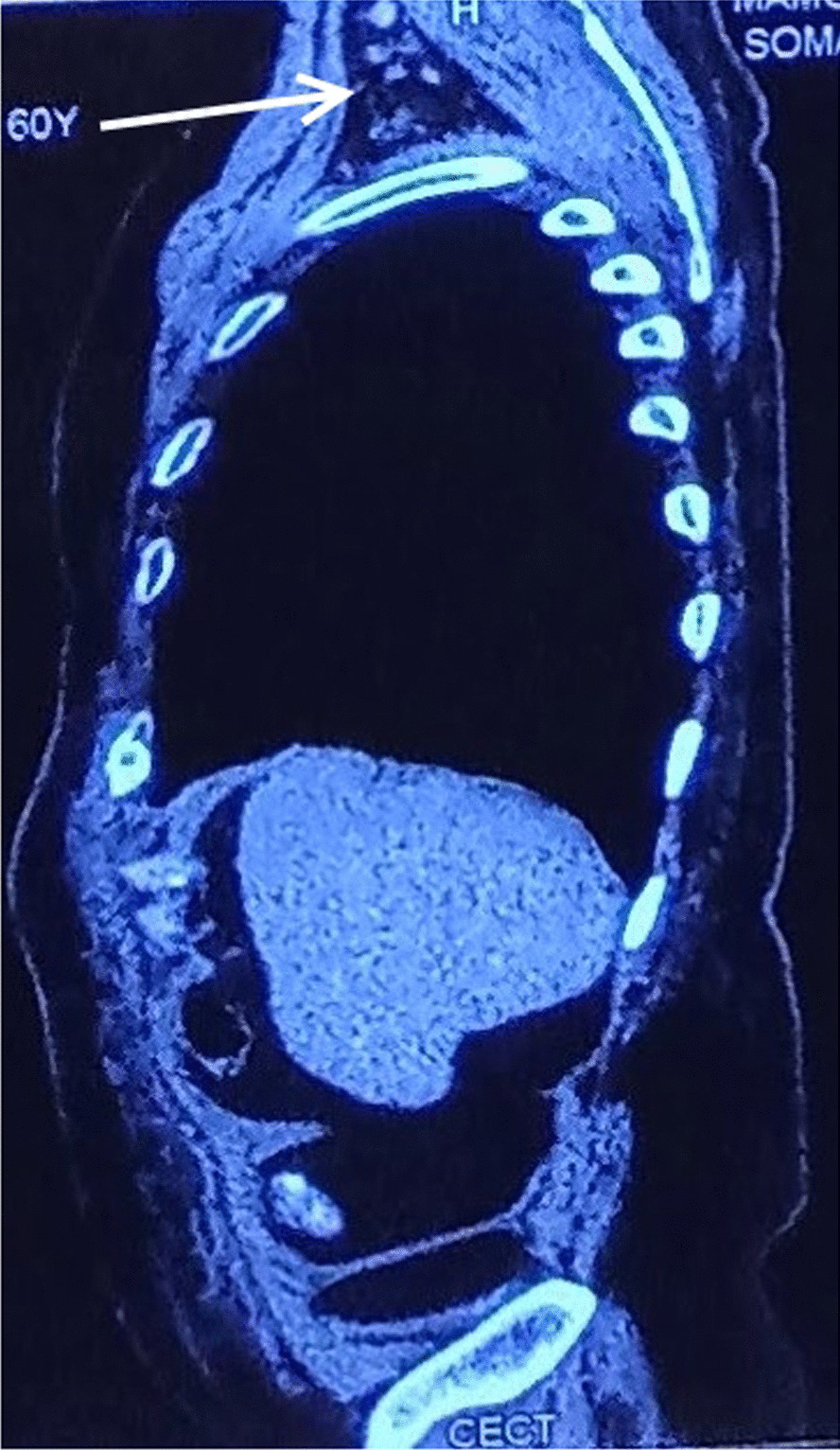
CECT Sagittal section showing right axillary lymph node metastasis (white solid arrow). The liver shows no metastatic lesions

**Fig. 3:**
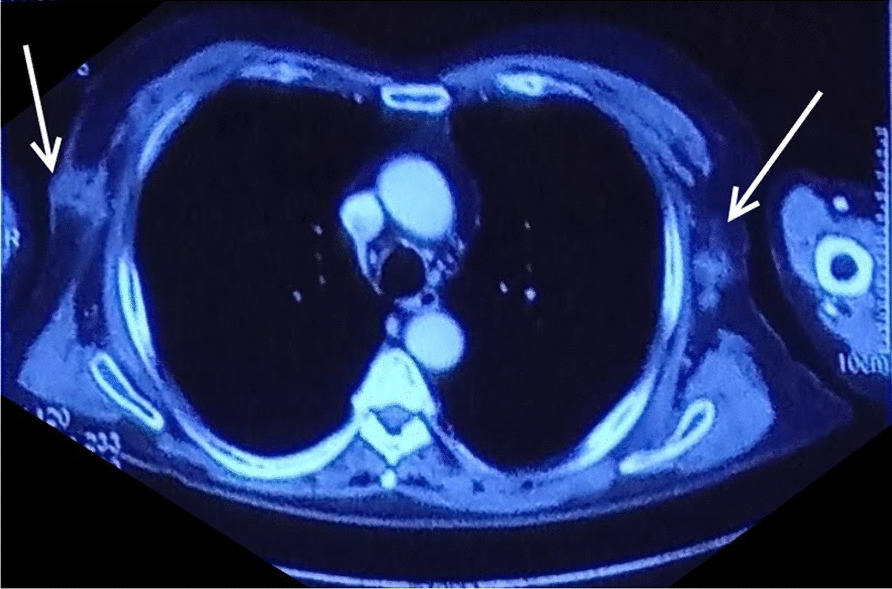
CECT Axial section showing bilateral axillary lymph nodes with invasion of right lymph node metastatic mass into the skin of right axilla (white solid arrows)

The patient was lost to follow-up thereafter and then presented to us almost 18 months after the operation with the complaint of bilateral axillary painless swellings for 3 months. Fine needle aspiration cytology from the bilateral axillary swellings was suggestive of metastatic adenocarcinoma. CT of the abdomen and chest was suggestive of well-defined soft tissue lesions in bilateral axilla measuring 4 × 4 cm on the left side and 6 × 4 cm (Figs. 1, 2, 3). PET scan of the patient was suggestive of bilateral axillary FDG positive deposits. After an extensive discussion with the oncologist, the patient was offered the option of bilateral axillary lymphadenectomy followed by chemoradiotherapy. The patient underwent bilateral axillary lymphadenectomy (Level I, II and III) and right sided wide local excision of skin and subcutaneous tissue because of fixity of lymph nodes to the skin.

**Fig. 4:**
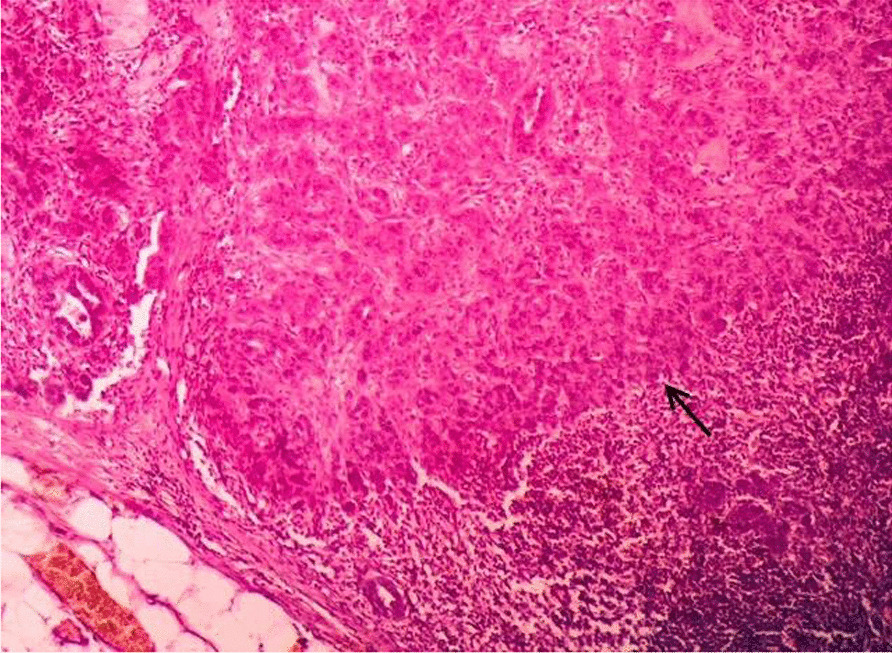
Histopathology Photomicrograph (Haemotoxylin and Eosin X 200) shows deposits of adenocarcinoma in the lymph node (solid black arrow)

**Fig. 5:**
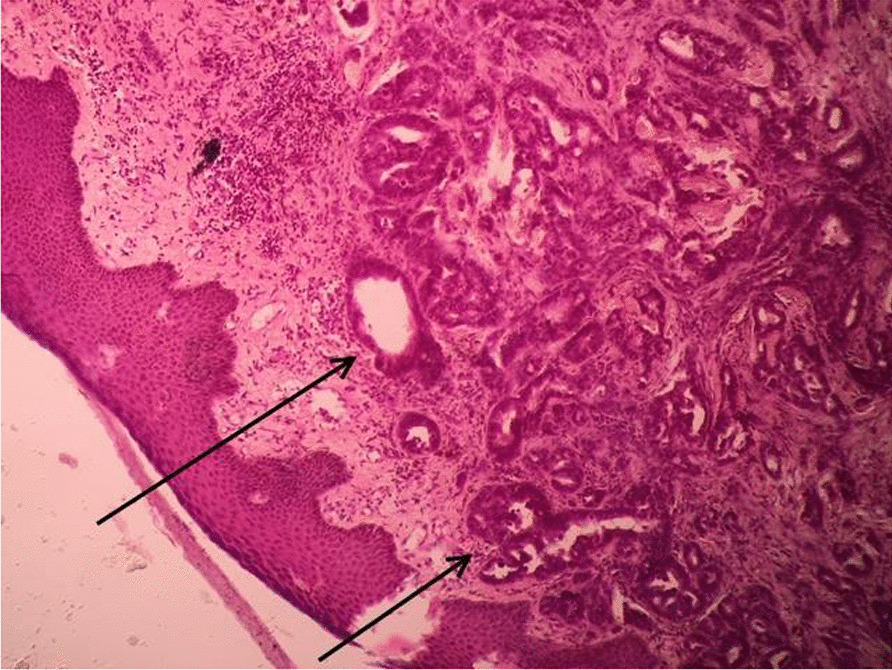
Histopathology Photomicrograph (Haemotoxylin and Eosin X 200) shows metastatic deposits of adenocarcinoma in the skin (solid black arrows)

**Fig. 6:**
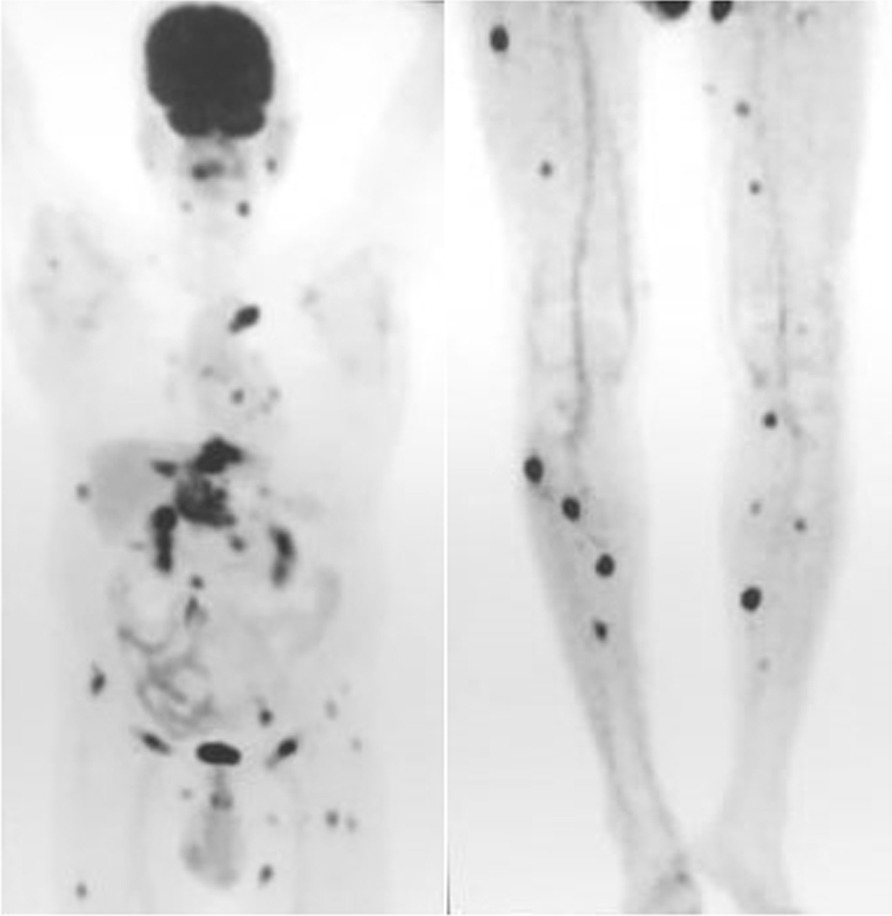
PET scan done after 6 months of bilateral axillary lymphadenectomy shows widespread metastasis. FDG avid lesions are visualized in the right sublingual, left pre-auricular, bilateral paraspinal lymph nodes, left internal mammary, bilateral hilar, subcarinal lymph nodes and left pleura and lower lobe. Metastasis to left lobe of liver, anterior abdominal wall at the previous operative site, omentum, peripancreatic, left external iliac, bilateral inguinal lymph nodes, bilateral paraspinal muscles, right intercostal muscles, bilateral gluteal muscles and multiple lower limb muscles is visible

During the post-operative period, the patient developed a seroma in the right axilla, which was managed by serial aspiration and compression. Histopathological examination (HPE) of the specimen from the left side identified 15 lymph nodes, out of which 10 showed metastatic adenocarcinoma with perinodal extension and presence of perineural and vascular invasion. 3 out of 11 lymph nodes on the right side showed adenocarcinoma deposits with peri nodal extension along with fibrosis and involvement of skin (Figs. 4, 5). After 3 months, the patient developed bilateral upper limb lymphoedema, which was managed by compression therapy and garments. The patient was advised to undergo a PET scan at the 6 month follow up visit. The scan revealed that the disease had spread to distant sites in the body. These included left lobe of liver, anterior abdominal wall, peripancreatic region, left external iliac and bilateral inguinal lymph nodes and multiple muscles throughout the body (Fig. 6). He was further advised radiotherapy/ chemotherapy after consultation from the oncologist at our institute, but he refused any further treatment. One month later, the patient succumbed to the disease.

## Discussion and conclusions

Gallbladder cancer is an aggressive malignancy, with most patients presenting in stage III/IV disease. 5-year survival rates for stage 0 is 80%, stage I is 50%, stage III is 7% and stage IV is 4% or less [10]. The usual sites of metastasis are the liver followed by the peritoneum [7]. Z’graggen et al. did a retrospective analysis of 10,925 patients who underwent laparoscopic cholecystectomy; 37 of them had unsuspected gallbladder cancer. Out of these, 5 patients (14%) had port site metastasis of the cancer irrespective of the stage of the primary cancer [9]. Metastasis to extra abdominal distant organs is rare and lung is the most common organ [11]. Nevertheless, metastasis to the heart [12], isolated metastasis to the breast [11] and simultaneous breast and ovarian metastases [13] have been reported in literature. Laparoscopic port site recurrence of gallbladder cancer was first reported by Drouard et al. [14] and Gornish et al., [15] independently, in the year 1991. A case of concurrent port site and axillary lymph node metastasis 12 weeks after laparoscopic cholecystectomy [16] and development of isolated unilateral axillary nodal metastasis 3–4 months after resection of abdominal port site metastatic recurrence have also been reported [6]. The evaluation of a patient with port site metastatic recurrence must be done meticulously. An attempt to retrieve records of the prior surgical procedure should be made. It is important to know the HPE report of the cholecystectomy specimen. Whether a protective bag or endo-bag was used during the procedure or not should be inquired for whenever possible. In the event of gallbladder rupture, the risk of seeding of the peritoneal cavity by tumour cells is increased [17]. During a laparoscopic cholecystectomy, if spillage occurs due to rupture of gallbladder when no protective bag is used, many surgeons will proceed with the resection of extraction port site/ all port sites when gallbladder carcinoma is suspected. On the contrary, this practice is not supported by literature [18, 19]. Peritoneal seeding of the tumour cells is not the only exclusive mechanism responsible for port site metastasis. Immune responses, creation of pneumoperitoneum, wound contamination and surgical methods have all been hypothesized to contribute to development of port site metastasis. Researchers have advocated stringent compliance to oncologic principles, meticulous use of modified surgical methods, pneumoperitoneum creation using hyperthermic and humidified CO_2_ to prevent occurrence of port site metastasis [20]. Furthermore, researchers have proposed that port site metastasis may possibly occur because of presence of circulating tumour cells at the time of penetrating surgical trauma. However, whether port site metastasis occurs or not depends on the patient’s immune response [21]. Zhu et al. proposed that excision of port sites only provides staging information that may eventually help in prognosticating patients of the risk of recurrence and is therefore beneficial but not necessary [17].

Gallbladder carcinoma usually spreads by direct extension into the liver and porta, via locoregional lymph nodes, by peritoneal seeding and by haematogenous routes [22–27]. It has also been known that carcinoma of gallbladder can spread along nerves and via the biliary tract [26]. On very rare occasions, carcinoma of the gallbladder may present as a long-standing gigantic gallbladder mass with preserved fat planes with organs in the near vicinity [28].

Possible mechanisms of spread of primary liver malignancy have been described in literature. The tumour which occupies the upper part of the right hepatic lobe may course through the lymphatic vessels to reach the lymph nodes on the upper surface of the diaphragm, mediastinal or parasternal lymph nodes [29, 30]. Malignant tumour cells may also spread from intercostal lymphatics to reach to the axillary lymph nodes. Other mechanisms of dissemination of tumour cells to the umbilicus include spread via a patent umbilical vein from portal venous channels; invasion of anterior peritoneum or by infiltration of the para-aortic glands [31]. The malignant cells may then drain from the subcutaneous lymphatic channels to the axillary lymph nodes [32].

Similar mechanisms of dissemination can be attributed for the spread of carcinoma gallbladder to the axillary nodes. The likelihood of spread of tumour from primary gallbladder cancer to the axilla by direct spread is extremely less. The more likely pathway for the spread of the tumour to the axilla is from the abdominal port site. However, it is more probable that the cause of abdominal port site metastasis is due to the systemic dissemination of the disease [9]. The axilla is a common draining site for many truncal malignancies, but gallbladder carcinoma usually spreads locally, and nodal metastasis occurs by involving the cystic, portal and peripancreatic nodes. Malignancies which may present with axillary lymph node metastasis include melanoma, carcinoma breast, malignant tumours of lung, ovary and stomach and therefore, must be excluded while evaluating the patient. Hu et al. also reported 2 cases of port site and distant metastasis detected by PET scan. Both patients had undergone laparoscopic cholecystectomy for an unsuspected gallbladder carcinoma. The report highlighted the important role of FDG-PET in follow-up of patients of gallbladder cancer after surgery [33].

Available literature shows only a handful of cases of axillary lymph node metastasis from a primary or recurrent gallbladder cancer. Johnson et al. had reported the first case of simultaneous occurrence of axillary lymph node metastasis along with abdominal wall port site metastasis occurring 12 weeks after the laparoscopic cholecystectomy. A T1 gallbladder carcinoma had been discovered during the surgical procedure [16]. Another report cited two cases of delayed development of isolated unilateral axillary nodal metastasis 3–4 months after complete resection of an abdominal wall port site metastatic recurrence from gallbladder cancer. Both the patients were diagnosed with adenocarcinoma gallbladder postoperatively by the histopathological examination report [6].

An R0 resection of the malignancy is the only viable option for effective therapy. However, the fact is that majority of the patients are unsuspected for gallbladder cancer and are diagnosed after laparoscopic cholecystectomy [9]. Laparoscopic cholecystectomy is an acceptable treatment for T1a gallbladder carcinoma. For more advanced lesions discovered after an initial laparoscopic cholecystectomy, a more extensive surgery like partial hepatectomy in the form of wedge or anatomical segment 4b and 5 resections with supraduodenal lymphadenectomy is recommended. Many patients require biliary reconstruction as well. This procedure has improved survival of patients. The 5-year survival of patients who underwent radical resection for cancer above stage I was 51% [34, 35].

Several authors are of the opinion that the satisfactory treatment of port site recurrence includes resection of the port site, even if it is for palliative intentions to avoid skin ulceration. It has also been postulated that the long-term outcomes of patients who undergo re-exploration after an unsuspected laparoscopic cholecystectomy and those who are adequately surgically treated at the time of intra-operative diagnosis of gallbladder carcinoma are same [36]. Even though the outcomes are the same, a re-operation is a more extensive procedure [6].

The present case shows the rare occurrence of isolated bilateral isolated axillary lymph node metastasis of gallbladder carcinoma after resection of the epigastric port site metastasis and adjuvant chemoradiotherapy, 15 months after re-exploration. Such a clinical presentation has not been described earlier in literature. Based on the few available reports which have been discussed; isolated bilateral axillary lymph node metastasis without any systemic metastasis can be treated with complete surgical resection i.e. Level I, II and III axillary lymphadenectomy. However, despite aggressive surgical management in a metastatic disease, the outcome still remains poor. Finally, the practice of delivering all gallbladders using an endo-bag or a retrieval bag after laparoscopic cholecystectomy cannot be overemphasized, especially in regions with high rate of GBC.

## Data Availability

All data generated or analyzed during this study are included in this manuscript.

## Abbreviations

GBC: Gallbladder carcinoma
ASR: Age standardized rate
ICMR: Indian Council of Medical Research
CECT: Contrast enhanced computed tomography
PET: Positron emission tomography
FDG: Fluorodeoxyglucose
HPE: Histopathological examination
